# Expansion of the Phenotypic and Genotypic Spectrum for *PRKAR1B* ‐Related Marbach–Schaaf Neurodevelopmental Syndrome: A Case Series

**DOI:** 10.1111/cge.70094

**Published:** 2025-10-29

**Authors:** Sebastian Burkart, Tarik Guzeloglu, Ana R. Soares, Irene Valenzuela, Eduardo F. Tizzano, David Gómez‐Andres, Laurent Pasquier, Marine Legendre, Camille Berges, Julien Thevenon, Marjolaine Gauthier, Caleb Heid, Elly Ranum, Joseph Shen, Michelle Frees, Michael W. Schmidtke, Caro Pilar, Christian P. Schaaf

**Affiliations:** ^1^ Institute of Human Genetics University Hospital Heidelberg Heidelberg Germany; ^2^ Medical Genetics Department Santo Antonio University Hospital Center Porto Portugal; ^3^ Clinical and Molecular Genetics Area, Vall D'hebron Hospital, Medicine Genetics Group Vall D'hebron Research Institute (VHIR) Barcelona Spain; ^4^ Pediatric Neurology, Vall D'hebron Institut de Recerca (VHIR) Hospital Universitari Vall D'hebron, Vall D'hebron Barcelona Hospital Campus Barcelona Spain; ^5^ Service de Génétique Clinique, CRMR Anomalies du Développement CLAD‐Ouest CRDI (Centre de Référence Déficiences Intellectuelles de Causes Rares), CHU Rennes Rennes France; ^6^ Service de Génétique Médicale—CHU Bordeaux Bordeaux France; ^7^ Genetic, Genomic and Procreation Department Grenoble Alpes University Hospital Grenoble France; ^8^ Thompson Center for Autism & Neurodevelopment University of Missouri Columbia Missouri USA; ^9^ Division of Genomic Medicine, Department of Pediatrics University of California Davis Sacramento California USA; ^10^ Department of Biological Sciences Wayne State University Detroit Michigan USA; ^11^ Institute of Human Genetics Heidelberg University Heidelberg Germany

**Keywords:** autism, case series, disability, genotype, neurodevelopmental disorder, pathophysiology, phenotype, *PRKAR1B*

## Abstract

Marbach–Schaaf neurodevelopmental syndrome (MASNS) is an ultra‐rare, monogenic disease caused by pathogenic variation in *PRKAR1B*, which codes for the R1β regulatory subunit of protein kinase A (PKA), a key effector of cAMP signaling within the nervous system. This work provides a comprehensive clinical description of 12 subjects with pathogenic *PRKAR1B* variants, including two individuals with a heterozygous deletion including *PRKAR1B*, supporting haploinsufficiency as a possible mechanism of disease. Phenotypic information was obtained by interview, using a systematic multi‐dimensional questionnaire. Besides expanding the evidence for established MASNS phenotypes like developmental delay, ID, ASD, pain insensitivity, as well as mild dysmorphisms, we broaden the clinical spectrum through the description of new and underreported findings, in particular increased body weight. In addition, the presence of genomic deletions suggests dosage sensitivity of *PRKAR1B*, demonstrating that both sequence and copy number variants should be considered in diagnostic testing. This work gives valuable insight into the pathophysiology of MASNS and sets a framework upon which to design future mechanistic studies of PKA signaling in brain development.

## Graphical abstract

Comprehensive clinical description of 12 subjects with pathogenic *PRKAR1B* variants, including two heterozygous deletions supporting haploinsufficiency as a possible mechanism of disease, providing valuable insight into the pathophysiology of MASNS and setting a framework upon which to design future mechanistic studies of PKA signaling in brain development.

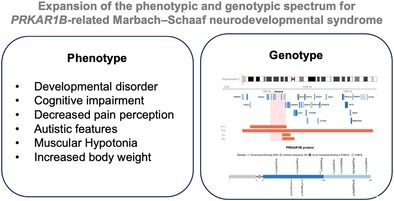

## 1 Introduction

Cyclic AMP‐dependent protein kinase A (PKA) is well known for its indispensable function in intracellular signaling across diverse tissues and organs. After activation by cyclic AMP (cAMP), it phosphorylates multiple downstream targets and is involved in pleiotropic biological processes [1]. Structurally, PKA is a heterotetrameric complex consisting of two regulatory and two catalytic subunits. The catalytic subunits exist mainly in two isoforms, whereas the regulatory subunits are subdivided into types I and II, each further distinguished into α and β subtypes. In the absence of cAMP, the catalytic subunits are autoinhibited by the regulatory counterparts. Upon the binding of cAMP molecules to the regulatory subunits' cAMP‐binding domains, the regulatory subunits dissociate from the complex, thereby releasing active catalytic subunits to phosphorylate downstream targets [2]. Each regulatory subunit, like PRKAR1B, is encoded by a distinct gene which shows tissue‐specific expression. The neuronal‐specific isoform of the regulatory subunits is R1β, which is encoded by the gene *PRKAR1B* [1, 2, 3, 4, 5, 6]. Besides the important role of *PRKAR1B* in health, there is increasing evidence for the role of *PRKAR1B* in disease. For example, a specific missense variant (c.149T>G p.Leu50Arg) in *PRKAR1B* has been associated with a hereditary late‐onset neurodegenerative phenotype [7, 8, 9]. Furthermore, several pathogenic germline variants in *PRKAR1B* cause the infantile‐onset autosomal‐dominant neurodevelopmental disorder Marbach–Schaaf neurodevelopmental syndrome (MASNS; OMIM #619680). MASNS is a rare disease characterized by neurodevelopmental delay, intellectual disability, and insensitivity to pain. To date, only 13 individuals with MASNS have been reported in the literature [10, 11]. This case series describes 12 additional individuals, expanding the genotypic and phenotypic spectrum of *PRKAR1B*‐related disorder.

## 2 Methods

### 2.1 Ethics and Data Collection

Upon receiving comprehensive information regarding the study's objectives, the patients' legal representatives provided written consent for the publication of clinical and genetic data. Identifiable data was pseudonymized. The patients' physicians and/or the caregivers were provided with a clinical questionnaire for a systematic and structured data collection (Data S1). Additional details were gathered by reaching out to the patients' parents or doctors directly.

### 2.2 Clinical Data Curation

After receipt of the completed clinical questionnaires (Data S1) from referring physicians, the data showed variable completeness and heterogeneous use of clinical terminology. To ensure consistency, we performed a systematic post hoc harmonization of key phenotypic variables. Obesity was defined in individuals with available age‐adjusted BMI values as a BMI above or within the 90–97th percentile, in accordance with established pediatric growth standards. In cases where BMI data were not available, the clinical assessment provided by the referring physician was used to determine the presence of obesity or increased body weight. Cognitive impairment was assumed for individuals who were reported to require any form of special education support. Autistic traits were considered present if explicitly described by the referring physician or reported by caregivers. Pain insensitivity was assessed based on documentation in the individual's medical history.

### 2.3 Genetic Data Curation

All variants were lifted over to GRCh38 and the MANE transcript (NM_001164760.2) using Alamut Visual Plus Software (v.1.12). For genomic deletion liftover, we used the lift over interface from Broad Institute (https://liftover.broadinstitute.org/). Decipher database (https://www.deciphergenomics.org/) was used for database screening for individuals with genomic deletions involving *PRKAR1B*. Respective phenotypic information was received from Decipher on July 18, 2025.

### 2.4 Statistical and Computational Analysis

Standard procedures of descriptive statistics were applied. Variables were illustrated using counts and percentages of the total cohort or on the total amount of cases with available data on that variable, unless stated otherwise. Missing data were not imputed. Percentages are rounded to whole numbers where appropriate. GnomAD variant allele frequency [12] and REVEL scores [13] were received from the Variant Effect Predictor (VEP) tool [14] after interfering all possible nucleotide changes within the coding sequence of *PRKAR1B* MANE transcript NM_001164760.2. Subsequently, mean values for the REVEL score and absolute gnomAD variant count at every amino acid position were calculated. For structural modeling, the 3D structure of the R1β subunit from the Protein Data Bank (ID of 4DIN; Chain B; https://doi.org/10.2210/pdb4DIN/pdb) was used. This structure represents the human protein of PRKAR1B bound to the mice protein of Pkaca (homolog of human PRKACA). Structural rendering and mutation placement were generated in PyMOL (Incentive Product v3.1.6.1; Schrödinger LLC). All analyses and illustrations were performed using R (version 4.4.0; 24.04.2024) environment for statistical computing and graphics (r‐project.org).

## 3 Results

In the following section, we provide a systematic description of the main clinical characteristics of the cohort. For detailed clinical and genetic information on the individual level, please refer to Table 1, which also includes the summarized review from two previously published *PRKAR1B* cohorts, thus comprising 25 individuals in total. For this study, a total of 12 individuals (eight males, four females) from unrelated, presumably healthy parents, each with a molecularly confirmed diagnosis of MASNS, were recruited.

**TABLE 1:** Clinical and genetic information for the individuals in the cohort.

Individual	#1	#2	#3	#4	#5	#6	#7
Sex	Male	Female	Female	Male	Female	Female	Male
Age (years)	9	56	4	21	9	5	4
DNA change (NM_001164760.2; GRCh38)	c.380C>T	c.1106A>C	c.1003C>T	c.1003C>T	c.1003C>T	c.734T>G	c.1003C>T
Protein change	p.(Ala127Val)	p.(Asn369Thr)	p.(Arg335Trp)	p.(Arg335Trp)	p.(Arg335Trp)	p.(Met245Arg)	p.(Arg335Trp)
Inheritance	De novo	Unknown (not inherited from mother)	De novo	De novo	De novo	NA	De novo
Family history	ALS in paternal grandmother; paternal cousin of mother with Down syndrome	Unremarkable	NA	Unremarkable	Unremarkable	A father's cousin and a mother's cousin with autism and intellectual retardation	Unremarkable
Pregnancy duration (weeks + days)	41 + 0	Normal	39 + 1	42 + 0	38 + 3	39 + 0	35 + 0
Maternal age at birth (years)	33	32	28	30	29	27	30
Paternal age at birth (years)	32	41	24	33	29	33	32
Fetal movement	Normal	Normal	Normal	Normal	Normal	Normal	Decreased
Type of delivery	SVD	SVD	SVD	SVD	SVD	C/S	C/S
Delivery complications	No	No	No	No	No	No	Yes (resuscitaion/CPAP/SCBU 5 days)
Birth weight (g)	3230	3160	2722	3200	3400	2850	2891
Birth length (cm)	49.5	NA	51.4	NA	51	NA	NA
Head circumference at birth (cm)	37	NA	36	35	34.5	NA	NA
Obesity/increased body weight (age‐ and sex‐matched reference percentile)	Yes	No	Yes (90–97 P.)	Yes	Yes	NA	NA
Lack of satiety	Yes	NA	NA	Yes (compulsive eating disorder)	Yes	No	NA
Hyperphagia	Yes	NA	NA	Yes	Yes	No	Yes
Other eating problems	NA	NA	Transient dysphagia	NA	No	No	NA
Pain tolerance	High	NA	High	High	Normal	Normal	High
Somatosensory system	NA	NA	NA	NA	Sensory seeking	Normal	NA
Temperature perception	NA	NA	NA	Impaired	Normal	Normal	NA
Formal cognitive evaluation (IQ or DQs)	Yes (Griffiths QGD 56)	Yes (severe ID)	NA	No	Yes (WPPSI‐III 71)	No	Yes (moderate ID)
Needs special education	Yes	Yes	NA	Yes	Yes	Yes	Yes
Attends mainstream school but needs resources	No	No	NA	No	Yes	No	No
Psychiatric history	NA	NA	NA	Self‐harm	NA	NA	NA
Attention deficit	NA	NA	NA	Yes	Yes	No	NA
Hyperactivity	NA	No	NA	Yes	Yes	No	Yes
Formal diagnosis of ADHD	NA	No	NA	NA	Yes	No	NA
Formal autism testing (ADOS, ADRI)	No	NA	NA	No	Yes (ADOS)	Yes	Yes
Meets diagnostic criteria of autism	No	No	NA	NA	Yes	Yes	Yes
Autistic features (without formal testing)	Yes	NA	Yes	Yes	Yes	Yes	NA
Gross motor skills	Impaired	NA	Impaired	Impaired	Impaired	Normal	Impaired
Fine motor skills	Impaired	NA	Impaired	Impaired	Impaired	Impaired	Impaired
Sitting without support (months)	7	Not applicable	10	10	9	7	11
Crawling (months)	9	Not applicable	20	19	14	9	14
Walking (months)	14	60	NA	24	19	NA	21
Motor skill regression	No	No	NA	No	No	No	Yes (12)
Age of first words (months)	12	Not applicable	NA	24	36	10	No
Age of combining words	No	No	NA	48 months	42 months	No	No
Fluent language	No	No	NA	No	No	No	No
Language regression	Yes (14 months)	No	NA	No	No	Yes (14 months)	Yes (13 months)
Hearing	Normal	No	NA	No	No	Normal	Fluid in ears awaiting further tests
Vision	NA	No	NA	No	Astigmatism	Normal	Astigmatism
Sleep abnormalities	Yes (snoring)	No	NA	No	NA	Yes (delayed sleep‐onsets)	Yes (sleep apnea, sleeps for long periods)
Seizures	No	Yes (generalized cryptogenic seizures at 5‐month‐old) Four drugs required	NA	No	No	No	Yes (no‐staring episodes for long periods)
Dyspraxia/apraxia	NA	NA	Yes (apraxia)	Yes (dyspraxia)	Yes	No	NA
Hypotonia	Yes	Yes	Yes	Yes	No	No	Yes
Feeding difficulties	No	No	Yes	No	Yes	No	Yes
Laboratory evaluation abnormalities	No	No	NA	No	NA	No	NA
MRI brain	Normal	No	Normal	Idiopathic neuroepithelial cyst in the left atrial region	Normal	No	Normal
EEG	Normal	NA	Normal	NA	NA	No	Normal
Congenital organ malformations	No	No	NA	No	No	No	No
Genitourinary anomalies	Not described	NA	NA	Small penis	No	No	No
Timing of puberty	NA	NA	NA	NA	NA	Not applicable (4 years)	NA
Skeletal anomalies	No	Osteoporosis	NA	No	No	No	No
Other abnormalities	No	Chronic leukemia LGL type	NA	NA	No	No	NA
Ears	Low‐set	NA	NA	NA	Normal	Normal	NA
Eyes	Subpalpebral fold	NA	NA	Epicanthus	Normal	Normal	NA
Hyper‐ or hypotelorism	Telecanthus	NA	NA	Almond‐shaped eyes with slight upward slant of the palpebral fissures, epicanthal folds, slight eversion of the distal third of the lower eyelid	Normal	Normal	NA
Nose	Hypoplastic columella, small nostrils, bulbous tip	NA	NA	Low nasal bridge	Upturned nose	Normal	NA
Philtrum	Short and a bit wide	NA	NA	Short philtrum	Short philtrum	Normal	NA
Mouth	Cupidbow upper lip, small diastema	NA	NA	NA	Thin upper lip	Normal	NA
Other	Mildly rough hair, umbilicated nipples	NA	NA	NA	NA	No	NA

Individual	#8	#9	#10	#11	#12	Summary	Marbach et al. [11] (summarized)	Marbach et al. [10] (summarized)
Sex	Male	Male	Male	Male	Male	8 males, 3 females	5 males, 1 females	3 males, 4 females
Age (years)	15	4	NA	3	21	—	—	—
DNA change (NM_001164760.2; GRCh38)	c.845T>C	c.904G>A	c.547G>T	chr7:290653–741467	chr7:193199–1816229	—	—	—
Protein change	p.(Ile282Thr)	p.(Val302Met)	p.(Asp183Tyr)			—	—	—
Inheritance	De novo	de novo	Unknown (not inherited from mother)	Unknown (not inherited from mother)	De novo	—	—	—
Family history	Congenital cardiopathy in the uncle of the mother	Unremarkable	Unremarkable	Brother with autism	Unremarkable	—	—	—
Pregnancy duration (weeks + days)	27 + 0	40 + 5	38 + 0	40 + 0	37 + 0	—	—	—
Maternal age at birth (years)	24	29	32	31	41	—	—	—
Paternal age at birth (years)	33	32	30	33	39	—		
Fetal movement	Normal	Normal	Decreased	Normal	Normal	Decreased (2/12)	—	—
Type of delivery	C/S	Vaginal vacuum delivery	SVD	SVD	C/S	—	—	—
Delivery complications	Yes (materno‐fetal infection and hyaline membrane disease)	No	No	No	Transient jaundice	—	—	—
Birth weight (g)	1075	3090	2950	2920	2891	—	—	—
Birth length (cm)	36	51	48	50.8	48.26	—	—	—
Head circumference at birth (cm)	25.5	36	34	35.6	NA	—	—	—
Obesity/increased body weight (age‐ and sex‐matched reference percentile)	Yes (90–97 P.)	Yes (90–97 P.)	NA	No	No	6 out of 12	2 out of 6	—
Lack of satiety	No	Yes	No	No	No	4 out of 12	—	—
Hyperphagia	No	Yes	No	No	No	5 out of 12	1 out of 6	—
Other eating problems	No	Taking too large of bites	NA	Limited food acceptance	No	—	—	—
Pain tolerance	High	High	No	Normal	Normal	6 out of 12	3 out of 6	5 out of 7
Somatosensory system	NA	Sensory seeking	Normal	Normal	Normal	Sensory seeking (2/12)	—	Hypersensitivity (1/7)
Temperature perception	Normal	Normal	No	Normal	Normal	—	—	Normal (7/7)
Formal cognitive evaluation (IQ or DQs)	Yes	NA	No	NA	Yes (very mild–normal ID)	6 out of 12; severe–mild	—	—
Needs special education	Yes	Yes	Yes	Yes	Yes	11 out of 12	6 out of 6	7 out of 7
Attends mainstream school but needs resources	Yes	Yes	Yes	NA	Yes	5 out of 12	—	—
Psychiatric history	No	NA	NA	NA	Self‐harm	2× self‐harm	—	—
Attention deficit	No	NA	No	No	Yes	3 out of 12	5 out of 6	7 out of 7
Hyperactivity	No	NA	No	No	Yes	4 out of 12	5 out of 6	7 out of 7
Formal diagnosis of ADHD	No	No	No	No	Yes	2 out of 12	5 out of 6	1 out of 7
Formal autism testing (ADOS, ADRI)	No	Yes (ADOS)	No	Yes	No	—	—	—
Meets diagnostic criteria of autism	No	Yes	No	Yes	Yes	6 out of 12	6 out of 6	1 out of 7
Autistic features (without formal testing)	No	Yes	No	NA	Yes	—	—	
Gross motor skills	NA	Impaired	Impaired	Impaired	Normal	Impaired 8/12	Impaired (6/6)	Impaired (4/7)
Fine motor skills	Impaired	Impaired	Impaired	Impaired	Normal	Delayed in 10/11	Impaired (6/6)	Impaired (7/7)
Sitting without support (months)	NA	6	10	7	6	Mean value: 8 months	—	—
Crawling (months)	NA	11	16	7	NA	Mean value: 14 months	—	—
Walking (months)	17	17	28	13	14	Mean value: 23 months	—	—
Motor skill regression	No	No	No	No	No	Yes (1/12)	Yes (1/6)	Yes (1/7)
Age of first words (months)		10	3 years	NA	12	Mean value: 25 months	—	—
Age of combining words	24 months	18 months	No	NA	NA	Mean value: 30 months	—	—
Fluent language	No	Yes	No	NA	Yes	2 out of 12		1 out of 7
Language regression	No	No	NA	NA	No	3 out of 12	Speech delay (5/6), speech regression in 2/6	1 out of 7
Hearing	No	No	No	NA	Chronic otits media recurrent	Intact among all	Intact among all	Intact among all
Vision	Astigmatism, hyperopia	No	Normal	No	Myopia	Astigmatism (3/12)	—	Myopia, strabism
Sleep abnormalities	No	No	No	No	No	3 out of 12	4 out of 6	4 out of 7
Seizures	Yes (monomorphic partial seizures, febrile seizures at 14, 22, and 27 months)	No	No	No	Yes (six febrile seizures)	4 out of 12	1 out of 6	No
Dyspraxia/apraxia	NA	Unsure	Yes	No	NA	4 out of 12	6 out of 6	2 out of 7
Hypotonia	NA	Yes	Yes	No	No	7 out of 12	3 out of 6	2 out of 7
Feeding difficulties	No	No	Yes	No	No	4 out of 12	—	2 out of 7
Laboratory evaluation abnormalities	No	NA	No	NA	No	No	—	—
MRI brain	Small calcification in the right caudate nucleus	Normal	Normal	No	No	—	—	—
EEG	Left hemispherical slow focus (wake + sleep), broad slow waves with a steep front‐facing right	Normal	No	No	NA	—	—	—
Congenital organ malformations	No	NA	No	No	No	—	—	—
Genitourinary anomalies	No	NA	No	No	No	—	—	—
Timing of puberty	Normal	NA	NA	NA	Early (8–9 years) ended with 16–18	—	—	—
Skeletal anomalies	No	No	No	No	No	—	—	—
Other abnormalities	No	No	No	No	No	—	—	—
Ears	Helix anomaly	NA	NA	Normal	Normal	—	—	—
Eyes	Epicanthus	NA	Epicanthus	Normal	Deep set eyes, lower set eyebrows	—	—	—
Hyper‐ or hypotelorism	NA	NA	NA	Normal	No	—	—	—
Nose	Bulbous tip, hypoplastic columella	NA	Slightly upturned	Normal	Prominent, wider nasal tip, low columella	—	—	—
Philtrum	NA	NA	Short	Normal	Short philtrum	—	—	—
Mouth	Thin upper lip, everted lower lip, high arched palate	NA	Thin lips	Normal	Microstomia mild	—	—	—
Other	NA	NA	Retrognathy	NA	Malar hypoplasia, mild prognathism	—	—	—

### 3.1 Clinical Description of the Patient Cohort

The age structure of the cohort at last clinical evaluation ranged between 3 and 56 years. All growth parameters for term‐delivered patients fall within the normal range. There was no evidence of complications during pregnancy or delivery, except for two individuals: #7 required resuscitation at birth, was stabilized with CPAP, and admitted to the neonatal intensive care for 5 days. #8, the only preterm delivery (27th week), had a maternal–fetal bacterial infection and hyaline membrane disease, requiring ventilatory support via intubation. Individuals #3, #5, #7, and #10 faced feeding difficulties during the neonatal period, with Individual #7 being initially tube‐fed before transitioning to breastfeeding. Individual #12 showed prolonged jaundice.

### 3.2 Neurodevelopment Phenotype

11/12 individuals (except #12) had global developmental delay (HP:0001263), which is a key symptom of MASNS. For individuals with available data, the mean age of free walking was 23 months (ranging 13–60 months), and the mean age for the first words spoken was 20 months (ranging 13–36 months). Eight individuals exhibited impaired gross motor skill development, while 10 were reported to have underdeveloped fine motor skills. In Individual #7, delays in both gross and fine motor skills were initially observed, but progress was noted over time. Notably, except for Individuals #9 and #12, there was no individual able to speak fluently, and only two out of the other 10 subjects were able to combine words (mean age 33 months). Furthermore, Individual #9 required extensive speech therapy to address echolalia and enhance conversational speech. Language and motor regression were present in (3/12) and (1/12) individuals, respectively. The earliest time of onset of regression was 12 months for motor and at 13 months for language skills in Individual #7.

All individuals (12/12) were described to have cognitive impairment, needing special education or support. Only four individuals were formally diagnosed with intellectual disability, but standardized testing data were only available for Individuals #1 (IQ 56) and #5 (IQ 71). The severity of the intellectual disability varied, with Individual #12 showing very mild–normal cognitive functioning, #1 showing mild (HP:0001256), #7 showing moderate (HP:0002342), and #2 showing severe intellectual disability (HP:0010864). 5/12 individuals were able to attend mainstream school with resources.

### 3.3 Behavioral Characteristics

While most individuals (7/9) manifested autistic traits (HP:0000729), such as hand flapping and poor social interaction, formal diagnosis of autism‐spectrum disorder (ASD) based on standardized testing was confirmed in five individuals. For the other individuals, no formal test results or data were available. 4/9 individuals exhibited hyperactivity and/or attention deficit, and two individuals received a formal diagnosis of attention deficit hyperactivity disorder (ADHD). Behavioral profile data are captured in Table 2.

**TABLE 2:** Behavior profile.

Individual	Hyperactivity	Underactivity	Stubbornness	Temper tantrums	Aggression	Controlling and manipulative behavior	Compulsivity	Anxiety	Social withdrawal	Difficulty with change in routine	Awareness of danger	Self‐harm
#1	3	NA	4	5	5	NA	4	NA	5	NA	NA	3
#2	NA	NA	NA	NA	NA	NA	NA	NA	NA	NA	NA	NA
#3	2	3	1	2	1	1	2	3	5	3	1	1
#4	5	1	5	4	3	2	4	4	2	5	1	3
#5	5	2	5	2	2	3	5	2	1	4	1	1
#6	1	1	2	2	1	1	1	3	3	1	2	1
#7	4	2	2	1	1	1	5	1	3	1	1	4
#8	1	1	1	1	1	1	1	2	1	4	1	1
#9	1	1	2	2	1	1	1	1	2	3	3	1
#10	1	1	4	4	2	1	2	1	1	1	1	1
#11	2	2	2	2	2	2	2	2	2	2	2	1
#12	1	4	2	2	1	1	2	4	4	2	3	2

*Note*: 1 = not present, 2 = rarely present, 3 = sometimes present, 4 = frequently present, 5 = always present.

### 3.4 Neuromuscular Phenotype

Muscular hypotonia (HP:0001252) was present in 7/11 individuals, with three subjects showing a more severe phenotype with remarkable perinatal muscular hypotonia. Individual #9 was the only one showing symmetric hyperreflexia in the lower extremities. Dys‐ or apraxia (HP:0002186) was present in 4/7 individuals, with an additional suspicion of dyspraxia in one patient.

Epilepsy (HP:0001250) or seizure‐like episodes were documented in 4/11 individuals. Individual #2 was recorded to experience epileptic encephalopathy at the fifth month of life, necessitating a regimen of four antiepileptic medications for management. Individual #8 experienced febrile seizures at 14, 22, and 27 months, followed by a monomorphic partial seizure without accompanying fever. Individual #12 had several febrile seizures. In addition, Individual #7 was reported to experience daily staring episodes, which had become less frequent over time and currently required no pharmacological intervention.

For the majority of the subjects who received brain magnetic resonance imaging (MRI) (9/12) and electroencephalography (EEG) (5/8), assessments yielded normal findings. However, an idiopathic neuroepithelial cyst was identified in the left atrial region of Individual #4, while Individual #8 exhibited a small calcification in the right caudate nucleus. Additionally, the EEG of Individual #8 demonstrated a left hemispheric slow focus (both when awake and asleep) and broad slow waves with a steep front‐facing slope in the right hemisphere.

Different unspecific sleep abnormalities were noted in 3/10 individuals. Individual #1 was observed to snore. While it was noted that Individual #6 struggled to fall asleep, Individual #7 tended to sleep for extended periods and presented with sleep apnea.

There was no evidence for recurrent neuro‐sensory phenotypes. Especially, hearing was found to be unaffected in most individuals upon evaluation. However, mild ophthalmologic phenotypes were described in some individuals, such as astigmatism (HP:0000483; Individuals #5, #7, and #8). Furthermore, Individual #7's mother raised concerns regarding a potential limitation in his peripheral vision, even though he had not received targeted diagnostic evaluation.

### 3.5 Pain Insensitivity

A variable degree of decreased pain perception (HP:0007328) was noticed in 6/11 individuals. A markedly high pain tolerance was seen in Individual #7, who had never been noted to respond to pain and had never been observed crying by his parents. In addition to high pain tolerance, Individual #4 also experienced impaired thermoregulation. Auto‐ and hetero‐aggressive behaviors (HP:0000718) were recorded in Individuals #4 and #12. Individuals #5 and #9 were known to seek sensory stimulation.

### 3.6 Obesity

In our cohort, six of nine individuals were classified as obese or overweight (> 90 percentile) (HP:0001513). No weight data was available for three individuals. Abnormal eating behavior (HP:0100738) like lack of satiety and hyperphagia was observed in 4/9 and 5/10, respectively. Individual #4 showed compulsive eating behavior. Individual #9 demonstrated signs of an intense obsession with food. For Individual #7, pica was documented.

### 3.7 Dysmorphic Features and Other Rare Phenotypes

Dysmorphic features did not indicate a recognizable facial gestalt for *PRAKR1B*‐related phenotype, but included unspecific features, such as short philtrum (HP:0000322), low nasal bridge (HP:0005280), and epicanthal fold (HP:0000286). Individual #3 presented with dysphagia. Notably, there were no instances of congenital organ malformation among the subjects.

### 3.8 Incidental Comorbidities

In addition to the primary disease manifestations, Individual #2 was diagnosed with chronic leukemia (LGL type) and osteoporosis. There was no evidence for other malignancies in the cohort; thus, this finding is regarded as a secondary diagnosis rather than part of the *PRKAR1B*‐related phenotype.

## 4 Genotype

**FIGURE 1:**
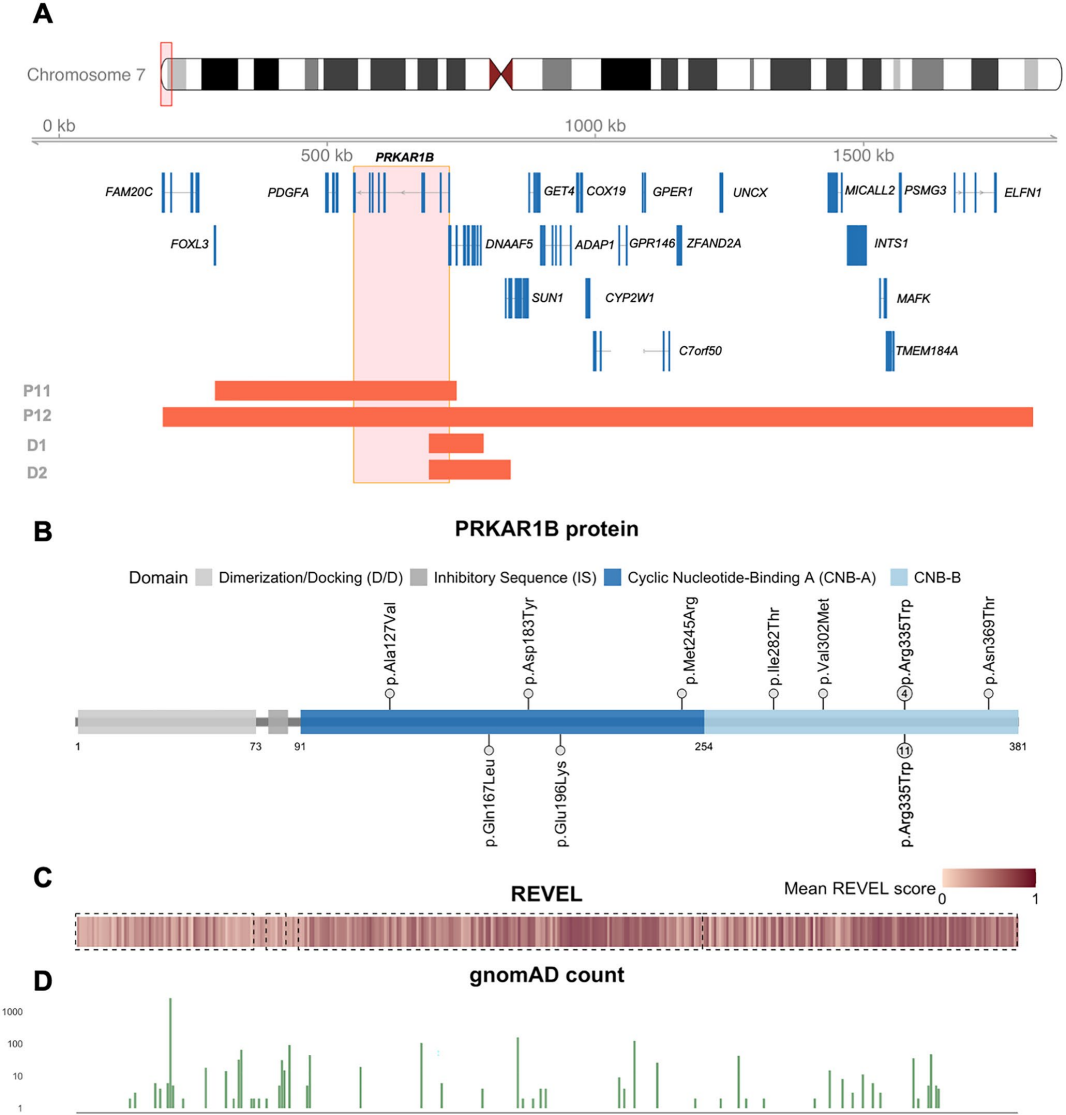
(A) Illustration of the genomic segment deletions of Individuals #11 (P11) and #12 (P12) and Decipher ID 547437 (D1) and 560256 (D2) including protein‐coding genes. For autosomal recessive disorders associated with *DNAAF5* (primary ciliary dyskinesia 18 #614874), *FAM20C* (Raine syndrome #259775), *INTS1* (NDCAGF #618571), and *GET4* (CDGIIY #620200), there was no evidence for a pathogenic variant on the remaining allele. For *FOXL3*, *PDGFA*, *SUN1*, *ADAP1*, *COX19*, *CYP2W1*, *GPR146*, *GPER1*, *ZFAND2A*, *UNCX*, *MICALL2*, *ELFN1*, *TMEM184A*, *MAFK*, *PSMG3*, and *C7orf50*, there is no associated OMIM phenotype. Based on this, we hypothesize that most, if not all, of our cohort's neurodevelopmental phenotype can be attributed to the *PRKAR1B* gene deletion and the resulting *PRKAR1B* haploinsufficiency. (B) Illustration of the protein structure of PRKAR1B based on crystal structure based on *PRKAR1B* transcript NM_001164760.2. Causative *PRKAR1B* variants are illustrated in their respective amino acid position (upper side of the protein scheme). Already reported missense variants are illustrated on the lower side of the gene scheme. Color coding reflects protein domains with cyclic nucleotide‐binding domains A and B. (C) Heatmap illustrating the mean REVEL score for every amino acid position of the PRKAR1B protein. Mean values for every amino acid position were calculated based on precomputed REVEL scores received from dbNSFPv5.0a. Dashed rectangles indicate localization of cyclic nucleotide‐binding domains A and B. (D) Bar plot illustrating the absolute allele count (log10 transformed *y*‐axis) for single nucleotide variants of *PRKAR1B* in healthy control individuals from gnomAD v4.1.0 across protein amino acid positions.

Individuals in this study exhibited a diverse range of PRKAR1B variants, including whole‐gene deletions (chr7:290653–741467 and chr7:193199–1816229) expanding the genotypic spectrum of MASNS (Figure 1A). We queried multiple public databases for other individuals carrying a small genomic deletion involving PRKAR1B, and we identified two individuals from the Decipher database with a corresponding deletion (P.547437: chr7:689554–841622 with intellectual disability and P.560256: chr7:689554–791274 with global developmental delay). The location and the involved genes of the deletions are shown in Figure 1A.

Besides the individuals with the deletions, all subjects carried missense variants, four of whom carried the previously described c.1003C>T p.(Arg335Trp) variant. This cohort includes variants which had not been previously reported (Figure 1B). All variants were confirmed *de novo* whenever parental testing was available. Parental testing was limited to one or neither parent in Individuals #2, #6, #10, and #11. All missense variants are located within the cyclic nucleotide‐binding domain A or B of the *PRKAR1B* gene (Figure 1B). These domains show higher mean REVEL scores (Figure 1C) and are more intolerant to variation as indicated by gnomAD alternative allele counts (Figure 1D). 3D structure localization of the missense variants is illustrated in Figure S1.

## 5 Discussion

With this case series, we provide in‐depth phenotypic and genotypic insight into *PRKAR1B*‐related Marbach–Schaaf neurodevelopmental disorder. Clinical data were obtained from 12 newly recruited individuals, bringing the total number of reported individuals for this ultra‐rare disorder to 25 [10, 11]. This cohort distinguishes itself from the previously described cohorts by offering insights into a larger, more varied group of individuals with diverse genetic variants from different ethnicities and ages.

Marbach–Schaaf syndrome is characterized by a highly variable and relatively nonspecific phenotype. Common features include developmental delay, intellectual disability, autism‐spectrum disorder, pain insensitivity, and mild dysmorphisms. This cohort expands the evidence regarding the variable expressivity of these traits, especially regarding ASD. In total, the ASD diagnosis frequency is at least 13 out of 25 (~52%). Hypotonia was more prevalent in this cohort compared to previous reports at least 58% (7/11) and ~38% (5/13), respectively. Decreased pain perception is one of the few specific features associated with MASNS. High pain tolerance was noted in several individuals, which aligns with findings previously reported. In particular, three patients carrying the c.1003C>T p.R335W variant seemed to demonstrate higher pain tolerance, consistent with the observations from the other 11 patients carrying the same variant. Anyway, decreased pain perception was also noticeable in a substantial number of individuals with other variants. Along with accumulating data from our cohorts, there is increasing evidence of *PRKAR1B's* critical role in pain perception. This is further supported by the finding that mice bearing a loss‐of‐function mutation in *PRKAR1B* exhibit diminished nociceptive pain perception [15].

Our cohort identified enrichment for increased body weight and obesity (6/9 individuals, 66%) compared to the global reference for children and adolescents aged 5–19 (20%) [16]. Additionally, some of our patients displayed symptoms of reduced satiety, hyperphagia, and overstuffing, contributing to increased body weight. Due to this finding, we suggest that perturbations in *PRKAR1B* may be causally related to obesity. Furthermore, a missense variant (p.R115K) in *PRKAR1B* was identified in a cohort of obese individuals, where it was suspected to alter lipoprotein profiles with favorable outcomes regarding alcoholic fatty liver disease [17]. Additionally, altered expression of the paralogous gene *PRKAR2B* negatively correlates with BMI levels [18]. Despite the limited evidence for a functional connection between *PRKAR1B* variants and increased body weight, our findings suggest that obesity might be a relevant phenotype in Marbach–Schaaf syndrome. As evidenced by some of our patients who display symptoms such as reduced satiety, hyperphagia, and overstuffing, contributing to increased body weight, it can be hypothesized that variants in *PRKAR1B* may influence eating behavior. Nevertheless, additional investigation is required to elucidate the relationship between variations in *PRKAR1B* and an individual's weight status. As a future perspective, systematically collected longitudinal body weight data and observation of eating behavior might provide insight into the clinical effects of *PRKAR1B* on body weight regulation. In clinical patient care, the knowledge of increased obesity risk will help to establish preventive measures, which are essential to avoid secondary complications.

This cohort provides a more diverse genotypic spectrum than previous publications, including different missense variants affecting various amino acid residues within the cyclic nucleotide‐binding A and B domains. We identified individuals with a *PRKAR1B* whole‐gene deletion within a small genomic segment deletion, including two individuals from this cohort as well as two individuals from the Decipher database (Figure 1A). Besides *PRKAR1B*, there is no established autosomal‐dominant trait associated with the genes affected by the genomic segment deletions. For autosomal recessive disorders associated with *DNAAF5* (primary ciliary dyskinesia 18 #614874), *FAM20C* (Raine syndrome #259775), *INTS1* (NDCAGF #618571), and *GET4* (CDGIIY #620200), there was no evidence for a pathogenic variant on the remaining allele. For *FOXL3*, *PDGFA*, *SUN1*, *ADAP1*, *COX19*, *CYP2W1*, *GPR146*, *GPER1*, *ZFAND2A*, *UNCX*, *MICALL2*, *ELFN1*, *TMEM184A*, *MAFK*, *PSMG3*, and *C7orf50*, there is no associated OMIM phenotype. Based on this, we hypothesize that most, if not all, of our cohort's neurodevelopmental phenotype can be attributed to the *PRKAR1B* gene deletion and the resulting *PRKAR1B* haploinsufficiency. These cases provide valuable evidence for haploinsufficiency as a potential pathomechanism of *PRKAR1B‐related* Marbach–Schaaf syndrome.

Interestingly, the individuals with the whole‐gene deletions (Individuals #11 and #12) show a milder phenotype with mainly autistic traits, relatively normal pain perception, milder cognitive impairment, and better neurodevelopmental outcomes compared to the individuals carrying missense variants (Table 3). Consistent with this observation, the aggregated disease burden, calculated as the sum of the core clinical features (cognitive impairment, hypotonia, increased body weight, decreased pain perception, and autistic features), was lower in the deletion carriers (median: 1.5) compared to the missense group (median: 3). Intellectual disability and global developmental delay were also described in the two deletion cases from Decipher, but in‐depth phenotypic information for these individuals is lacking. More cases with *PRKAR1B* full gene deletions are needed to comprehensively capture the phenotype of *PRKAR1B* haploinsufficiency.

**TABLE 3:** Differences in molecular subgroups (missense vs. deletion).

	Variant	Present	Absent	Odds ratio (missense vs. deletion)	95% CI (low–high)	*p* (Fisher's exact)
Increased body weight	Missense	6	1	OR not estimable (due to zero cell)	0.56–1023.6	0.0833
	Deletion	0	2			
Decreased pain perception	Missense	6	3	OR not estimable (due to zero cell)	0.27–238.5	0.1818
	Deletion	0	2			
Cognitive impairment	Missense	9	0	OR not estimable (all affected)	0.07–302.7	1
	Deletion	2	0			
Autistic features	Missense	6	2	OR not estimable (due to zero cell)	0.04–64.0	1
	Deletion	1	0			
Hypotonia	Missense	7	2	OR not estimable (due to zero cell)	0.43–452.0	0.1091
	Deletion	0	2			

*Note*: Odds ratios and 95% CIs were derived from Fisher's exact test (conditional likelihood). In tables with zero cells, odds ratios were not estimable, but exact confidence intervals are reported.

Our findings suggest that haploinsufficiency of *PRKAR1B* is sufficient to cause a clinical phenotype consistent with a dosage‐sensitive mechanism. On the molecular level, pathogenic missense variants may lead to haploinsufficiency by reducing levels of functional PRKAR1B protein [11]. However, as *PRKAR1B* is part of a heterotetrameric protein complex, missense variants might cause disruptive interference with the residual function of the wild‐type protein [2]. Structural modeling indicates that the missense variants are predominantly located on the surface of the regulatory subunit, where they are positioned to affect intersubunit interfaces rather than core cAMP‐binding structures. This surface localization supports a potential dominant‐negative mechanism through aberrant interactions within the heterotetrameric protein complex. This is in line with the observation that missense variants are associated with a more severe phenotype, indicating a dominant‐negative effect. Functional studies on the effect of gene deletions and missense variants are necessary to confirm haploinsufficiency and/or dominant‐negative effects as pathomechanisms.

So far, complete penetrance of the disorder is expected, as there is no evidence of a pathogenic variant inherited from an apparently healthy parent. In fact, we are not aware of familial cases of MASNS.

This case series contributes to cumulative evidence for *PRKAR1B*‐related MASNS, providing additional insight that haploinsufficiency might be a possible pathomechanism. This demonstrates that both sequence and copy number variants should be considered in diagnostic testing for autism and global developmental delays. We provide a systematic collection of phenotypic and genotypic data for this ultra‐rare condition, expanding our understanding of the gene's role in neurodevelopmental disorders. This contributes to the establishment of genotype–phenotype correlations, which are highly important to provide prognostic information and to potentially guide future therapeutic strategies.

## 6 Limitations and Challenges

This study has limitations and faces methodological challenges, which are important to consider for interpretation. First, in some cases, a clear distinction between perinatal complications and disorder‐related manifestations remains challenging, particularly given the possibility that *PRKAR1B* dysfunction may itself predispose to pregnancy/delivery complications. Additionally, the presence of seizures or epilepsy in some individuals may confound assessments of developmental and cognitive outcomes. Second, clinical data were collected by multiple referring physicians, introducing the potential for inter‐observer variability bias, which may contribute to inconsistencies in phenotypic reporting. The reliance on post hoc reverse phenotyping—where clinical features are interpreted retrospectively following a genetic diagnosis—introduces the risk of confirmation bias. This may partly explain the observed variability and mild, often nonspecific, dysmorphic features reported in affected individuals. The integration of quantitative, instrumented assessment metrics represents an important future direction to enhance reproducibility and cross‐cohort comparability.

Substantial efforts were made to overcome these limitations and challenges by establishing a systematic and structured data collection questionnaire and conducting comprehensive post hoc harmonization (Data S1). All referring physicians are experts in their fields, further highlighting the quality of our clinical data. Thus, we consider our study reliable and generalizable in the context of the aforementioned important limitations.

## Author Contributions

Conceptualization: S.B., T.G., C.P., and C.P.S. Data collection: C.P., T.G., A.R.S., I.V., E.F.T., D.G.‐A., L.P., M.L., C.B., J.T., M.G., C.H., E.R., J.S., M.F., M.W.S., and C.P.S. Methodology: T.G., S.B., C.P., and C.P.S. Formal analysis and investigation: S.B. Writing – original draft preparation: S.B. and T.G. Writing – review and editing: C.P. and C.P.S. Supervision: C.P.S.

## Conflicts of Interest

The authors declare no conflicts of interest.

## Supporting information


**Data S1:** cge70094‐sup‐0001‐DataS1.pdf.

**Figure S1:** Structural localization of the *PRKAR1B* missense variants. The 3D structure of the PRKAR1B protein was received from Protein Data Base (PDB ID: 4DIN, Chain B). Localization of missense variants is highlighted on the protein structure, illustrating their predominant localization at the surface rather than within the cAMP‐binding pockets. Structural rendering and mutation placement were performed in PyMOL (v.3.1.6.1; Schrödinger LLC).

## Data Availability

The data that support the findings of this study are available in the Supporting Information of this article.
