# Supplementary material for: Expansion of the Phenotypic and Genotypic Spectrum for PRKAR1B ‐Related Marbach–Schaaf Neurodevelopmental Syndrome: A Case Series
Source: Clin Genet. 2025 Oct 29;109(4):679–96. doi: 10.1111/cge.70094 (PMC12958008; doi:10.1111/cge.70094)
Supplement: Supplementary file 1 — Data S1: cge70094‐sup‐0001‐DataS1.pdf. [file CGE-109-679-s001.pdf]

Clinical Questionnaire PRKAR1B

1 - General information, pregnancy and neonatal period

Name	
Date of birth	
Sex	
Ethnicity	
PRKAR1B Variant (NM_001164760) and mode of inheritance	
**pregnancy and birth history**	
Pregnancy Duration	
Maternal age (at birth)	
Paternal age (at birth)	
Fetal movement (normal, decreased)	
Fetal position at time of delivery	
Type of delivery (C/S, SVD, etc.)	
Complications after delivery	
Birth Weight	
Birth length	
Head circumference at birth	
**Neonatal period**	
Hypotonia	
Feeding difficulties	
Other abnormalities	
**Family History**	
# of siblings (males, females)	
Grandparents alive?	
Family history of developmental delay, intellectual disability, muscular problems, etc.)	

2 – Somatic, neurological, and psychological features

**General growth (measurements and age at the time of measurement)**	
Head circumference (last evaluation)	
Weight (last evaluation)	
Height (last evaluation)	
**Dysmorphic features**	
Ears	
Eyes (epicanthal folds etc.)	
Hyper- or hypotelorism	
Palpebral fissure length	
Nose (upturned, short, etc.)	
Philtrum (short, prominent, smooth, etc.)	
Mouth (thin lips, palate, etc.)	
Hands (please include size/percentile)	

Feet (please include size/percentile)	
Other (please specify)	
**Congenital organ malformations** (e.g. heart defects, intestinal malformations, etc.)	
**Genitourinary anomalies** (e.g. cryptorchidism, micropenis, enlarged labia etc.)	
Timing of puberty (premature, delayed)	
Menarche (which age, amenorrhea)	
**Skeletal anomalies** (e.g. hip dysplasia, scoliosis, etc.)	
**Eating behavior**	
Lack of satiety	
Hyperphagia	
Other eating problems	
**Neurologic examination**	
Pain tolerance	
Somatosensory system	
Temperature perception	
Gross motor skills	
Fine motor skills	
Dyspraxia/Apraxia	
Hypotonia	
Muscular strength	
Other abnormalities (please specify)	
**Seizure history** (incl. seizure type, frequency, type and duration of seizure medication, etc.)	
**Hearing** (e.g. hearing impairment)	
**Vision** (e.g. strabismus, nystagmus, myopia etc.)	
**Sleep abnormalities** (e.g. sleep apnea, abnormal circadian rhythm, etc.)	
**Developmental milestones (motor)** (age in months)	
Sitting without support	
Crawling	
Walking	
Motor skill regression?	
**Developmental milestones (language)**	
Age of 1st words (months)	
Age of combining words	
Fluent language	
Language regression?	
**Autism**	
Any formal testing (ADOS, ADRI)	

Meets diagnostic criteria of autism	
Autistic features (without formal testing)	
**ADHD:**	
Attention deficit	
Hyperactivity	
Formal diagnosis of ADHD (attention deficit hyperactivity disorder), at which age?	
**Psychiatric History:**	
**Cognitive evaluation**	
Formal cognitive evaluation (IQ or DQs)	
Needs special education	
Attends mainstream school but needs resources	
**Other anomalies**	
**Laboratory evaluation/metabolic, endocrine anomalies**	
**Other anomalies not mentioned above**	
**Diagnostic studies**	
MRI brain	
EEG	

3 - Behavioral Profile

➔ Please assign a number from 1-5 (1 = not present; 2 = rarely present, 3 = sometimes present, 4 = frequently present, 5 = always present)

**Trait**	**1 - 5**
Hyperactivity	
Underactivity	
Stubbornness	
Temper tantrums	
Aggression	
Controlling and manipulative behavior	
Compulsivity	
Anxiety	
Social withdrawal	
Difficulty with change in routine	
Awareness of danger	
Self-harm (e.g. skin picking)	
**Other traits (please specify)**	**1 - 5**
