# Supplementary figures and images for: Expansion of the Phenotypic and Genotypic Spectrum for PRKAR1B ‐Related Marbach–Schaaf Neurodevelopmental Syndrome: A Case Series
Source: Clin Genet. 2025 Oct 29;109(4):679–96. doi: 10.1111/cge.70094 (PMC12958008; doi:10.1111/cge.70094)

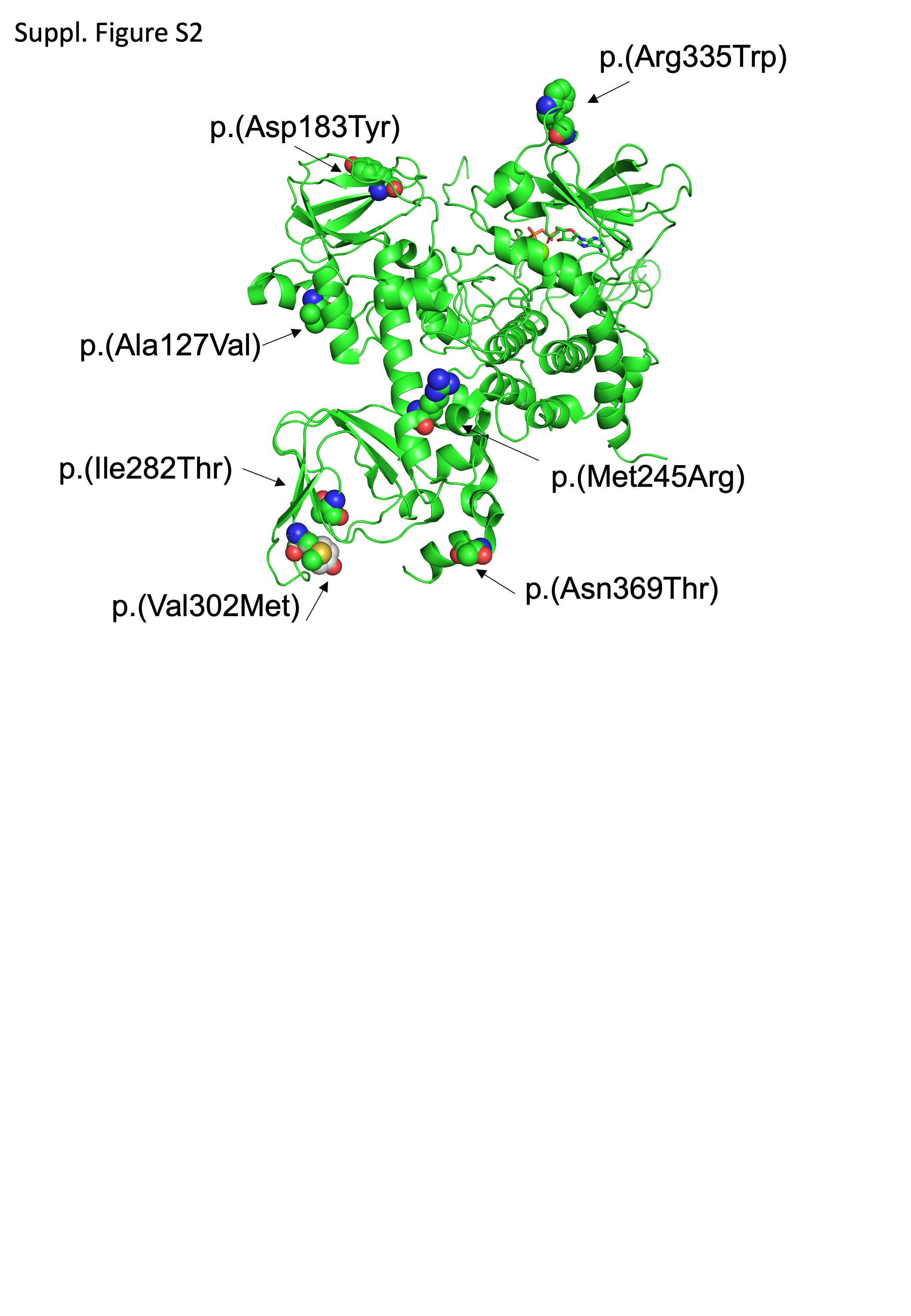

Supplement: Supplementary file 2 — Figure S1: Structural localization of the PRKAR1B missense variants. The 3D structure of the PRKAR1B protein was received from Protein Data Base (PDB ID: 4DIN, Chain B). Localization of missense variants is highlighted on the protein structure, illustrating their predominant localization at the surface rather than within the cAMP‐binding pockets. Structural rendering and mutation placement were performed in PyMOL (v.3.1.6.1; Schrödinger LLC). [file CGE-109-679-s002.tiff]
